# Oxidative Stress Genes in Diabetes Mellitus Type 2: Association with Diabetic Kidney Disease

**DOI:** 10.1155/2021/2531062

**Published:** 2021-09-02

**Authors:** Athanasios Roumeliotis, Stefanos Roumeliotis, Fotis Tsetsos, Marianthi Georgitsi, Panagiotis I. Georgianos, Aikaterini Stamou, Anna Vasilakou, Kalliopi Kotsa, Xanthippi Tsekmekidou, Peristera Paschou, Stylianos Panagoutsos, Vassilios Liakopoulos

**Affiliations:** ^1^Division of Nephrology and Hypertension, 1st Department of Internal Medicine, AHEPA Hospital, School of Medicine, Aristotle University of Thessaloniki, 54636 Thessaloniki, Greece; ^2^Department of Molecular Biology and Genetics, Democritus University of Thrace, 68100 Alexandroupolis, Greece; ^3^1st Laboratory of Medical Biology-Genetics, School of Medicine, Aristotle University of Thessaloniki, 54124 Thessaloniki, Greece; ^4^Department of Microbiology, AHEPA Hospital, School of Medicine, Aristotle University of Thessaloniki, 54636 Thessaloniki, Greece; ^5^Division of Endocrinology and Metabolism-Diabetes Center, 1st Department of Internal Medicine, AHEPA University Hospital, Department of Medicine, Aristotle University of Thessaloniki, Thessaloniki, Greece; ^6^Department of Biological Sciences, Purdue University, West Lafayette, 47907 Indianapolis, USA; ^7^Department of Nephrology, School of Medicine, Democritus University of Thrace, 68100 Alexandroupolis, Greece

## Abstract

Diabetic type 2 patients compared to nondiabetic patients exhibit an increased risk of developing diabetic kidney disease (DKD), the leading cause of end-stage renal disease. Hyperglycemia, hypertension, oxidative stress (OS), and genetic background are some of the mechanisms and pathways implicated in DKD pathogenesis. However, data on OS pathway susceptibility genes show limited success and conflicting or inconclusive results. Our study is aimed at exploring OS pathway genes and variants which could be associated with DKD. We recruited 121 diabetes mellitus type 2 (DM2) patients with DKD (cases) and 220 DM2, non-DKD patients (control) of Greek origin and performed a case-control association study using genome-wide association data. PLINK and EIGENSOFT were used to analyze the data. Our results indicate 43 single nucleotide polymorphisms with their 21 corresponding genes on the OS pathway possibly contributing or protecting from DKD: SPP1, TPO, TTN, SGO2, NOS3, PDLIM1, CLU, CCS, GPX4, TXNRD2, EPHX2, MTL5, EPX, GPX3, ALOX12, IPCEF1, GSTA, OXR1, GPX6, AOX1, and PRNP. Therefore, a genetic OS background might underlie the complex pathogenesis of DKD in DM2 patients.

## 1. Introduction

Patients with a history of diabetes mellitus type 2 (DM2) compared to patients without DM2 exhibit increased risk of diabetic kidney disease (DKD), diabetic retinopathy, peripheral vascular disease, and peripheral neuropathy which are the major micro- and macrovascular complications of the disease [1]. DKD affects 20-30% of patients suffering from DM2 and is the leading cause of end-stage kidney disease (ESKD) in most developed countries. Many pathways, mechanisms, and genes have been implicated in DKD development and progression including hyperglycemia, hypertension, inflammation, and oxidative stress (OS) [2, 3].

OS along with the related genes is a pathway leading to DKD development as well as a mechanism related to major pathways responsible for diabetic damage [4]. OS is defined by increased reactive oxygen species (ROS), increased reactive nitrogen species (RNS), and reduced ability of the innate antioxidant defense system which eventually cause insulin resistance, DNA, lipid and protein damage, and gene expression alterations. OS is present and enhanced from the early CKD stages to dialysis and is associated with cardiovascular morbidity and mortality [5–7].

DM2-DKD heritability is multifactorial and heterogeneous. This implies that in populations of different ethnic background, a great number of gene polymorphisms and gene polymorphism combinations are implicated in DKD pathogenesis. Most DKD gene studies refer either to DM1 or to Afro-American, Asian, and American populations, but none has genotyped the Greek population. Yet, DM1 compared to DM2 and American and Asian populations compared to European exhibit a different and distinct genetic basis. Thus, only genetic results in DM2-DKD including European populations may be comparable to our study results. In addition, many genome-wide association studies (GWASs) and meta-analyses are aimed at revealing those polymorphisms that increase the risk for DKD development in DM2. Despite numerous gene locus identification, the results of the studies are inconsistent and the identification of gene variants robustly associated with DKD are limited [8]. Our study is aimed at investigating which gene polymorphisms on the OS pathway are possibly associated with DKD in diabetic type 2 patients.

## 2. Patients and Methods

A total of 341 patients of Greek Caucasian origin suffering from DM2, according to ADA (https://www.diabetes.org/a1c/diagnosis), for at least 10 years were included in the study. Only patients with a long history of DM2 were included in the study in an effort to let microvascular complications of DM2, such as DKD, evolve. Inclusion and exclusion criteria have been described elsewhere [9]. Exclusion criteria included known cancer/chemotherapy treatment, obstructive uropathy, and family relation to an already recruited patient. The patients, both case and control groups, were followed in the outpatient clinic of the University General Hospital of Alexandroupolis. The case group consisted of 121 DM2 patients with DKD, diabetic retinopathy in fundoscopy, and persistent proteinuria (>0.5 g/day) or albuminuria (≥30 mg/day) (inclusion criteria for case group). The control group included 220 DM2 patients without DKD, no sign of diabetic retinopathy, no proteinuria (<0.15 g/day), normoalbuminuria (<30 mg/day), and an estimated glomerular filtration rate (eGFR) of above 60 mL/min/1.73 m^2^, calculated by the Chronic Kidney Disease Epidemiology Collaboration (CKD-EPI) equation [10]. Every patient had signed an informed consent in advance. The study protocol was approved by the Ethics Committee of the Scientific Board of the University Hospital of Alexandroupolis and was in accordance with the *Helsinki Declaration of Human Rights*.

## 3. Laboratory Measurements and Genotyping

Laboratory measurements were performed on morning blood and urine samples after an at least 10-hour fasting and genomic DNA was extracted from peripheral whole blood using the Qiagen Gentra Puregene kit (Qiagen, Hilden, Germany). The selection markers for the study were based on the pathophysiologic pathway of OS because it is considered a common pathophysiologic pathway for both DM2 and DKD. DNA was genotyped on the Illumina Human PsychArray-24 v.1.1 BeadChip, containing probes for 603,132 single nucleotide polymorphisms (SNPs), 559,921 of which are submitted to dbSNP and have received an rs number. The Infinium PsychArray BeadChip is very similar to the MetaboChip and other specialized chips by Illumina. They all share the same backbone, which is 271,000 SNPs from the Infinium Core-24 BeadChip and 277,000 markers from the Infinium Exome-24 BeadChip, with the capacity of investigating a variety of diseases. Their difference is approximately 50,000 SNPs that are included based on previous GWAS on related phenotypes, depending on the focus. Our study follows a candidate gene approach, focusing only on genes participating in the OS pathophysiologic pathway, which are indeed included in the BeadChip we used. For the analysis, standard GWAS quality control steps were followed as extensively described elsewhere [11]. More specifically, 1449 SNPs and their corresponding genes were studied: ANGPTL7, GPX7, DHCR24, CSDE1, PRDX6, NCF2, GLRX2, TPO, MPV17, GGCX, TTN, SGO2, AOX1, STK25, OXSR1, GPX1, GPR156, RNF7, ADIPOQ, ADIPOQ, SOD3, SPP1, NFKB1,SEPP1, GPX8, NME5, GPX3, ATOX1, DUSP1, GPX6, GPX5, TNF, AGER, VEGFA, GSTA7P, GSTA2, GSTA1, GSTA5, GSTA3, GSTA4, IPCEF1, SOD2, NUDT1, IL6, GTF2I, NCF1, CALU, NOS3, MSRA, EPHX2, CLU, SCARA3, OXR1, MBL2, SFTPD, PDLIM1, BNIP3, CAT, PRG3, PRDX5, RELA, CCS, MTL5, FOXM1, MGP, KRT1, GPX2, DUOX2, DUOXA1, DUOX1, NOX5, MT3, CYBA, ALOX12, CCL2, CCL5, EPX, MKS1, LPO, MPO, CYGB, GPX4, PRDX2, SIRT2, APOE, PNKP, SRXN1, PRNP, GSS, SGK2, PREX1, SOD1, TXNRD2, and DGKK because of their relation with OS pathway.

## 4. Statistical Analysis

Data were tested for normality using Kolmogorov-Smirnoff test in the IBM SPSS software (Statistical Package for Social Sciences, 18.0 for Windows, Chicago, IL, USA). Normally distributed continuous variables are reported as mean ± standard deviation (SD), whereas nonnormally distributed variables are reported as median. Patient characteristics were compared between case and control groups using chi-square test (*χ*^2^) test for categorical variables. *χ*^2^ was also used for single-marker association analysis in order to compare allele and genotype frequencies between the DKD and non-DKD DM2 patient groups. Our results were further assessed by performing permutation testing on the data, which is considered to be the gold standard in keeping the false-positive rate constant. For the purpose of this analysis, we used the PLINK software suite [12] and EIGENSOF*Τ* [13]. PLINK is used for genetic data analysis and operates on the digital representation of genetic data that have been produced by genotyping. It assumes a binary form of biallelic genetic variants and is capable of performing a multitude of statistical tests. A newer version of PLINK implements multithreading into the computationally intensive calculations that are needed for large-scale data that are generated from the latest technologies [12]. In addition, one of the most important obstacles in conducting reliable genetic analyses is overcoming confounding. A major source of confounding can be found in the naturally occurring differences between geographically distant populations, better known as population stratification. The EIGENSOFT software suite, which uses Principal Component Analysis, was used in order to analyze the genetic variation of genome-wide data [13]. Initially, significance was set for *p* value less than 5 × 10^−8^, which is the case in GWAS. As no SNP reached this significance, *p* value was set below 0.05.

## 5. Results

In this GWAS, we tried to discover possible associations between gene polymorphisms and DKD. Demographic, biochemical and clinical parameters of both case and control groups are shown in Table 1. Although no SNP achieved genome-wide statistical significance (*p* < 5 × 10^−8^), a statistically significant association for *p* < 0.05 was found for 43 SNPs and their corresponding genes. Some of these SNPs have been studied in the literature and have been either correlated with DKD presence or not reached statistical significance [11, 14–28]. The rest have not been reported yet.  Table 2 presents in order of statistical significance the SNPs and their corresponding genes that attained a *p* value of less than 0.05 and are possibly associated with DKD in DM2 patients of Greek origin. The minor allele responsible for the associated polymorphism, percentage of minor allele's presence in case and control groups, the major allele, *χ*^2^ values, and odds ratio (OR) along with confidence interval (CI) and standard error (SE) are also shown.

In the case that a particular SNP is only found in the control group and thus the percentage of the SNP in the case group is 0%, then the OR calculation is 0 and the SE approaches infinity. On the contrary, when a particular SNP is only found in the case study group and thus the percentage of the SNP in the control group is 0%, then the OR and SE cannot be calculated. When OR for a specific SNP is greater than 1, it means that this SNP possibly contributes to DKD development. On the other hand, an OR below 1 implies that the specific SNP could possibly exhibit a protective role against DKD development in DM2 patients.

In order of statistical significance the SNPs and their corresponding genes that possibly protect from DKD development in DM2 patients and attained a *p* value less than 0.05 include the following: in chromosome (chr.) 4 rs7685225-SPP1 (secreted phosphoprotein 1), in chr.2 rs1567919-TPO (Thyroid Peroxidase) and rs13431646-TPO, in chr.11 rs486584-CCS (Copper Chaperone for Superoxide Dismutase), in chr.4 rs2725236-SPP1 and rs6833161-SPP1, in chr.8 rs7824574-CLU (Clusterin), in chr.17 rs14309-ALOX12 (arachidonate 12-lipoxygenase), in chr.6 rs1293928-IPCEF1 (Interaction Protein for Cytohesin Exchange Factors 1), in chr.8 rs10087808-OXR1 (Oxidation Resistance 1), in chr.2 rs2465661-AOX1 (Aldehyde Oxidase 1), in chr.7 rs6947821-NOS3 (nitric oxide synthase 3), in chr.8 rs10108813-OXR1 and rs1503573-OXR1, and in chr.20 rs6052780-PRNP (Prion Protein). On the other hand, the SNPs and their genes that possibly lead to DKD development in DM2 patients and attained a *p* value less than 0.05 in order of statistical significance include the following: in chr.2 rs6588678-TPO, rs56307213-TTN (Titin) and rs72648987-TTN, rs9678469-TPO, rs72650006-TTN, and rs17532665-SGO2 (Shugoshin 2); in chr.7 rs7830-NOS3; in chr.10 rs45458497-PDLIM1 (PDZ and LIM domain 1); in chr.2 rs17448235-SGO2; in chr.8 rs11780592-CLU, rs72677233-TTN, and rs72646855-TTN; in chr.19 rs201633492-GPX4 (glutathione peroxidase 4); in chr.22 rs201971987-TXNRD2 (Thioredoxin Reductase 2) and rs737866-TXNRD2; in chr.2 rs6732480-TPO; in chr.8 rs2741335-EPHX2 (Epoxide Hydroxylase 2); in chr.11 rs3019593-MTL5 (Metallothionein-Like Protein 5); in chr.2 rs2048727-TPO; in chr.17 rs9904720-EPX (Eosinophil Peroxidase); in chr.5 rs2230303-GPX3 (glutathione peroxidase 3); in chr.5 rs8177413-GPX3; in chr.22 rs12106549-TXNRD2; in chr.6 rs6905523-GSTA7P (soluble glutathione serine transferase alpha 7*Ρ*); in chr.6 rs35062161-GPX6 (glutathione peroxidase 6); in chr.8 rs10109171-OXR1; in chr.6 rs72944451-GSTA4; and in chr.17 rs11652709-EPX.

From the aforementioned SNPs, the ones that have not been reported in the literature in correlation with diabetes or DKD are mentioned below. The TTN polymorphisms with statistical significance include rs56307213 (*p* = 0.002), rs72648987 (*p* = 0.002), and rs72650006 (*p* = 0.005), as well as rs72677233 (*p* = 0.019) and rs72646855 (*p* = 0.019) found only in the case group. These TTN SNPs possibly contribute to DKD development in DM2 patients. SGO2 rs17532665 (*p* = 0.005) and rs17448235 (*p* = 0.01) could be possibly leading to DKD development in DM2. PDLIM1 gene top hit found in our study was rs45458497 (*p* = 0,005), which could possibly exhibit a contributing role in DKD development in DM2 patients. CLU gene rs11780592 (*p* = 0.013) is possibly contributing to DKD development, and rs7824574 (*p* = 0.039) is possibly protecting from DKD occurrence in DM2 patients. CCS top hit was rs486584 (*p* = 0.019) which is possibly protective against DKD development in DM2 patients. Top hit polymorphism for GPX4 gene was rs201633492 (*p* = 0.019), identified only in the case group and possibly contributing to DKD development. TXNRD2 gene rs201971987 (*p* = 0.039) was identified only in the case study group while rs737866 (*p* = 0.020) and rs12106549 (*p* = 0.019) were found in both groups. These SNPs are possibly contributing to DKD development in DM2 patients. In our study, we also identified MTL5 rs3019593 (*p* = 0.033) possibly contributing to DKD development in DM2 patients. EPX gene rs9904720 (*p* = 0.035) and rs11652709 (*p* = 0.049) that gained statistical significance possibly contribute to DKD development in DM2 patients. Additionally, in our study, rs6905523 of GSTA7P pseudogene of the soluble glutathione serine transferases (GST gene family) and rs72944451 of GSTA4 have attained statistical significance (*p* = 0.041 and *p* = 0.049, respectively) and are possibly associated with DKD development. OXR1 gene top hit SNPs in our study included rs10087808 (*p* = 0.042), rs10108813 (*p* = 0.048), and rs1503573 (*p* = 0.049), which are possibly protective against DKD development, while rs10109171 (*p* = 0.044) is possibly involved in DKD development in DM2 patients. GPX6 gene rs35062161 attained statistical significance (*p* = 0.044) and is possibly associated with DKD development. PRNP gene top hit SNP in our study was rs6052780, which reached a *p* value of 0.049 and is possibly protective against DKD development. The rest of the SNPs have been reported in the literature in correlation with DKD and are further discussed.

## 6. Discussion

The increased incidence of DKD cannot be solely explained by traditional risk factors [2]. A number of SNPs and their corresponding genes have been associated with DKD in several populations and studies. The engulfment and cell motility 1 (ELMO1) gene has shown an association with DKD in a Japanese population [29], a finding verified in a review and meta-analysis including both DM1 and DM2 populations of different origin [30]. The sterol regulatory element-binding protein-1 gene (SREBPF-1) has been also found to contribute to DM2 and DKD in a French, obese population suffering from DM2 as well as in mice [31, 32]. Polymorphisms of the Transcription Factor 7-Like 2 (TCF7L2) gene have been associated with DM2 incidence in European and African-American populations, suffering from micro and macrovascular complications of DM2 including DKD [33, 34]. The C677T variant of the methylenetetrahydrofolate reductase (MTHFR) gene on the metabolic pathway of homocysteine has been associated with DKD presentation and progression in DM2 populations of south Indian [35] and Tunisian origin [36] and in a meta-analysis including several studies [30]. Polymorphisms in the nonmuscle myosin IIA gene (MYH9) have been associated with ESKD susceptibility in a European American population with DM2 [37]. Another important pathway involved in DKD is the renin-angiotensin-aldosterone system (RAAS). The variants of Met235Thr in angiotensinogen, T>C (-344) in aldosterone synthase and the G>A (-1903) in chymase genes, have been associated with DKD in an Asian population suffering from DM2 [38]. Yet, none of these genes are on the OS pathway. Our study is aimed at identifying those SNPs on the OS pathway, along with their corresponding genes, that reached a statistical significance of less than 0.05 and are possibly associated with DKD development.

Secreted phosphoprotein 1 (SPP1) gene resides on 4q22.1 region of chr.4 and encodes a protein known as osteopontin (OPN). This protein is a proinflammatory cytokine that upregulates phospholipase-C pathway and expression of interferon-gamma, interleukin-12, transforming growth factor-*β*, and fibronectin and downregulates interleukn-10. OPN is involved in tissue and bone remodeling, bone resorption, inflammation, fibrosis, obesity, acute kidney injury, acute glomerulonephritis [39], kidney stone formation [40], DM2, and diabetic nephropathy [40]. High glucose levels result in OPN production, which in turn promotes inflammation and extracellular matrix turnover, thus resulting in diabetic renal injury and diabetic nephropathy [15]. Additionally, OPN is a critical regulator of adipose tissue and is drastically upregulated in obesity, leading to inflammation, insulin resistance, atherosclerosis, DM2, and diabetic nephropathy [40]. In our study, rs7685225 (*p* = 0.001), rs2725236 (*p* = 0.26), and rs6833161 (*p* = 0.037) reached statistical significance and could possibly play a protective role from DKD development in DM2 patients.

Thyroid Peroxidase (TPO) gene resides on the 2p25.3 region of chr.2, encodes a membrane-bound glycoprotein, and plays a central role in thyroid gland function, OS pathway, and DM2 occurrence [41]. Specifically, thyroid hormone regulates mitochondrial gene expression and function in skeletal muscle and exerts a role in metabolic energy balance and subsequent obesity. Mitochondrial OS in skeletal muscle potentially contributes to increased lipid accumulation and impaired metabolism, which are both features of insulin resistance and diabetes type 2. In addition, in OS, reductions in T3-mediated transcription have been suggested to lead to complications related to diabetes [41]. Data associating specific TPO SNPs with DKD are not reported in the literature. Nevertheless, in our study, TPO SNPs that gained statistical significance were rs6588678 (*p* = 0.002), rs9678469 (*p* = 0.002), rs6732480 (*p* = 0.028), and rs2048727 (*p* = 0.035), which possibly contribute to DKD development in DM2 patients. On the contrary, rs1567919 and rs13431646 which were only identified in the control group and both attained a *p* value of 0.018 could possibly exhibit a protective role against DKD development.

Nitric oxidase synthase 3 (NOS3) gene is located on the 7q36.1 region of chr.7. Endothelial isoform of the NOS (eNOS), encoded by NOS3 gene, converts L-arginine to L-citrulline and releases nitric oxide (NO), which is involved in the regulation of renal and glomerular hemodynamics. Endothelial dysfunction serves as key event in the development and progression of diabetic vascular complications, inflammation, and fibrosis which eventually lead to diabetic nephropathy, hypertension, and coronary artery disease [14, 42]. Impaired eNOS activity increases OS activation and plays a central role in endothelial cell dysfunction at numerous levels, including enzyme uncoupling, posttranslational modifications, internalization, and decreased expression [43]. Reduced NO bioavailability exacerbates OS, further promoting endothelial dysfunction and injury [43]. The injured endothelial cells may then function as active signal transducers of metabolic, hemodynamic, and inflammatory factors that modify the function and morphology of the vessel wall and interact with adjacent cells. This may activate a cascade of inflammatory, proliferative, and profibrotic responses as DKD progresses [43]. NOS3 gene is suggested as a potential candidate gene to DKD, while other studies have not confirmed this finding [16, 17]. Most common SNPs associated with DKD include G894T (rs1799983), T-786C (rs2070744), rs7830, and 4b/a [16, 17]. In our study, top hit SNPs for NOS3 gene were rs7830 (*p* = 0.005) which is possibly associated with DKD development and rs6947821 (*p* = 0.048), which probably plays a protective role against DKD occurrence in DM2 patients.

Epoxide Hydroxylase 2 (EPHX2) gene resides on the 8p21.2-p21.1 region of chr.8 and encodes a protein member of the epoxide hydroxylase family, soluble epoxide hydroxylase (sEH). Arachidonic acid (AA) is metabolized by epoxygenase enzymes to biologically active epoxyeicosatrienoic acids (EETs) which exhibit an antihypertensive, anti-inflammatory role, preserve renal function, reduce OS activation, and increase insulin sensitivity [18, 44]. SEH is a cytosolic homodimer enzyme intracellularly composed of two domains [19]. The N-terminal domain hydrolyzes EETs to the less active dihydroxyepoxyeicosatrienoic acids (DHETs), thus limiting their effects, while the C-terminal domain hydrolyzes lipid phosphates [19, 44]. SEH inhibition has beneficial effects on renal function [18]. SEH deficiency in podocytes has been shown to preserve renal function and glucose control and mitigate hyperglycemia-induced renal injury and is associated with attenuated hyperglycemia-induced renal endoplasmic reticulum stress, inflammation, and fibrosis [44]. Inhibition of sEH prevents from renal interstitial fibrosis, and EPHX2 whole-body deficient mouse display reduced renal inflammation and injury [44]. Because of its central pathophysiologic role in disease states such as hypercholesterolemia, cardiac hypertrophy, diabetes, hypertension, ischemic stroke, cancer, arteriosclerosis, and pain, sEH is currently being investigated as a potential therapeutic target [19]. Polymorphism rs41507953 (K55R) has been implicated in coronary artery disease [20], while R287Q (rs751141) has been associated with lower risk of diabetic nephropathy in Chinese type 2 diabetic patients [21]. In our study, statistically significant rs2741335 (*p* = 0.031) is possibly involved in DKD development in DM2 patients.

Glutathione peroxidase 3 (GPX3) gene resides on 5q33.1 region of chr.5 and belongs to the selenoprotein gene family [23]. The glucosylated protein encoded by GPX3 gene belongs to the glutathione peroxidase family with 8 glutathione peroxidases (GPX1-8) identified to date [22]. The protein is found mainly in kidney and adrenal gland, but also in heart, lung, and cerebellum and protects cells and enzymes from oxidative damage, by catalyzing the reduction of hydrogen peroxide (H2O2), lipid peroxides, and organic hydroperoxides. GPX3 acts as a redox buffer for the discrimination between irrelevant and serious inflammatory stimuli [22]. GPX3 can prevent hydroperoxide-mediated activation of lipoxygenases (LOXs). Activation of LOXs amplifies any initial phagocyte response or otherwise activated ROS release and leads to a full inflammatory response if H2O2 is not immediately eliminated [22]. GPX3 prevents such signal amplification at membranes and resolves inflammatory responses [22]. GPX3 deficiency has been associated with inflammatory bowel disease, DM2, obesity, and cardiovascular disease [22, 24]. In the kidney, GPX3 probably protects podocytes from the malevolent role of OS in DKD [45]. GPX3 polymorphisms have been associated with increased risk of stroke, while rs8177409 has been associated with increased cardiovascular risk [24]. In our study, GPX3 rs2230303 (*p* = 0.036) and rs8177413 (*p* = 0.037) were possibly associated with increased risk of DKD development.

Arachidonate 12-lipoxygenase (ALOX12) or 12S type resides in 17p13.1 region of chr.17 [11]. ALOX12 is a human LOX, which belongs to a family of enzymes responsible for oxidative metabolism of polyunsaturated free fatty acids to generate lipid mediators including eicosanoids and lipoxins by inserting molecular oxygen into them [11]. ALOX12 has been implicated in pathways such as the polyols, hexosamines, protein kinase C, advanced glycosylation end-products, vasoconstriction, atherosclerosis, inflammation, and OS, as well as in albuminuria in DM2 (rs1126667) and diabetic nephropathy [11]. In the existence of hyperglycemia, ALOX12 also causes endothelial dysfunction and renal vasoconstriction, leading to renal injury and CKD progression [11]. Top hit for ALOX12 gene in our study was rs14309 which attained a *p* value of 0.040 and is possible protective against DKD development in DM2.

Interaction Protein for Cytohesin Exchange Factors 1 (IPCEF1) gene resides on region 6q25.2 of chr.6 and encodes a protein associated with DKD and OS in GWAS [25]. Among other related pathways, IPCEF1 binds and enhances cytohesin 2 activity, takes part in ADP-ribosylation factor 6 (Arf6) signaling events and OS, as it is implicated in peroxidase activity and oxygen transporter activity [26]. Statistical significant polymorphisms associated with DKD in a meta-analysis of DM1 patients were rs955333 and rs12523822 [25]. In our study, though, rs1293928 was the only SNP of the IPCEF1 gene that reached statistical significance (*p* = 0.041) and is possibly protective from DKD development.

The Aldehyde Oxidase 1 (AOX1) gene resides on 2q33.1 region of chr.2 and produces aldehyde oxidase, a molybdo-flavoenzyme, mainly expressed in liver, but also present in lungs, kidneys, and intestines. AOX1 is essential for podocyte function and structure [46]. AOX1 is characterized by broad substrate specificity, oxidizes aromatic aldehydes into carboxylic acids and hydroxylates various heteroaromatic rings [27, 46]. AOX1 is a candidate gene for amyotrophic lateral sclerosis [28], and upregulated enzyme levels have been implicated in xanthinuria, cancer, and diabetes through OS activation [46]. While the complete physiological role is still unclear, AOX1 enzyme exhibits an emerging role in drug metabolism and drug discovery [27, 46]. In our study, AOX1 rs2465661 (*p* = 0.046) could be possibly protective. However, as AOX1 gene is still under investigation and expressed in the kidneys, it is speculated that rs2465661 could possibly inhibit OS activation and eventually play a protective role for podocytes. Finally, since AOX1 is involved in drug metabolism through pathways other than CYP450, drugs to protect from DKD occurrence in DM2 patients may be developed in the future [46].

The rest of the genes investigated in our study, despite their association with OS, exhibit a lack of correlation with diabetes and DKD in the literature. These include, TTN, SGO2, PDLIM1, MTL5, CCS, GPX4, TXNRD2, CLU, EPX, GST-alpha class, Oxidation Resistance 1, GPX6, and PRNP.

Finally, genes and SNPs associated with DKD in other populations of DM2 patients were not confirmed in our study results. A reason for this discrepancy could be that a SNP may not have a significant effect on DKD, but it may be in close linkage disequilibrium with other causative mutations not yet identified and the allele associated with the real causative mutations varies across ethnic groups. Another possible explanation is that perhaps the polymorphism that affects the development of the disease is influenced by other genes and proteins collectively, which are all together dependent on the entire genetic background of the population, dietary habits, and environmental diversities. Last but not least, the relatively small sample size of our study may have served as a limitation, and thus, GWASs with larger population size are needed.

In conclusion, 43 polymorphisms in our study attained a *p* value less than 0.05 and are thus possibly contributing to or protecting from DKD development in DM2 patients of Greek origin. Implementation of a mathematical model or prognostic algorithm could assist in identifying those diabetic type 2 patients with a high risk of DKD occurrence. Despite our study's small sample size, the polymorphisms identified could also help further targeted investigation for the specific SNPs leading to DKD.

## Figures and Tables

**Table 1:** Patient demographic, biochemical, and clinical parameters according to group. DM2: diabetes mellitus type 2; DKD: diabetic kidney disease; HbA1c: hemoglobin A1c; HDL: high-density lipoprotein; LDL: low-density lipoprotein.

	Case group, with DM2 and DKD	Control group, with DM2 and without DKD	*p* value
Gender (males/females)	68/53	102/108	0.031
Age (years)	50.6 ± 10.9	52.98 ± 10.7	0.067
DM2 duration (years)	19.66 ± 8.46	17.15 ± 11.6	0.051
Body mass index (kg/m^2^)	31.73 ± 5.54	31.06 ± 5.74	0.43
Waist circumference (cm)	106.8 ± 17.12	103.96 ± 14.61	0.09
HbA1c (%)	7.53 ± 1	7.33 ± 1.2	0.18
Fasting blood glucose (mg/dL)	163 ± 73	150 ± 48	0.08
Triglycerides (mg/dL)	175 ± 116	154 ± 87	0.1
Total cholesterol (mg/dL)	168 ± 46	184 ± 41	0.006
HDL-cholesterol (mg/dL)	44 ± 14	51 ± 18	0.0002
LDL-cholesterol (mg/dL)	91 ± 40	102 ± 36	0.026

**Table 2:** The single nucleotide polymorphisms (SNPs) of the genes that attained a significant association with diabetic kidney disease in diabetic type 2 patients of Greek Caucasian origin. Polymorphisms are presented by level of significance based on increasing *p* value. The minor allele responsible for the SNP variant, the percentage of minor allele appearance in both case and control groups, and the major allele are shown. SNP: single nucleotide polymorphism; OR: odds ratio; CI: confidence interval; SE: standard error; NC: not calculable.

Chromosome	SNP-gene	Minor allele	% of minor allele in case group patients	% of minor allele in control group patients	Major allele	*χ* ^2^	*p* value	Odds ratio (OR) (CI: confidence interval)	Standard error (SE)
4	rs7685225-SPP1	C	25.21	37.27	T	10.28	0.001	0.567 (0.4-0.81)	0.1779
2	rs6588678-TPO	A	51.24	38.86	G	9.743	0.002	1.653 (1.2-2.27)	0.1616
2	rs56307213-TTN	G	4.959	1.136	C	9.385	0.002	4.539 (1.58-13.04)	0.5385
2	rs72648987-TTN	A	4.959	1.142	G	9.318	0.002	4.518 (1.573-12.98)	0.5385
2	rs9678469-TPO	G	17.36	9.545	A	8.818	0.002	1.99 (1.256-3.153)	0.2348
2	rs72650006-TTN	A	6.25	2.074	T	7.849	0.005	3.148 (1.356-7.307)	0.4296
2	rs17532665-SGO2	G	18.6	10.91	A	7.832	0.005	1.865 (1.2-2.9)	0.2251
7	rs7830-NOS3	A	40.91	30.23	C	7.937	0.005	1.598 (1.152-2.217)	0.1669
10	rs45458497-PDLIM1	C	4.545	1.142	T	7.861	0.005	4.124 (1.416-12.01)	0.5455
2	rs17448235-SGO2	G	18.6	11.47	A	6.561	0.01	1.763 (1.138-2.732)	0.2234
8	rs11780592-CLU	G	25	17.05	A	6.176	0.013	1.622 (1.105-2.381)	0.1957
2	rs1567919-TPO	T	0	2.273	G	5.582	0.018	0	Infinite
2	rs13431646-TPO	T	0	2.273	C	5.582	0.018	0	Infinite
2	rs72677233-TTN	T	1.24	0	C	5.479	0.019	NC	NC
2	rs72646855-TTN	C	1.24	0	T	5.454	0.019	NC	NC
11	rs486584-CCS	G	43.39	52.73	A	5.447	0.019	0.6871 (0.5011-0.9422)	0.1611
19	rs201633492-GPX4	A	1.24	0	G	5.479	0.019	NC	NC
22	rs201971987-TXNRD2	G	1.24	0	A	5.479	0.019	NC	NC
22	rs737866-TXNRD2	G	38.02	29.32	A	5.393	0.02	1.479 (1.062-2.059)	0.1688
4	rs2725236-SPP1	T	38.02	46.82	C	4.916	0.026	0.6967 (0.5059-0.9595)	0.1633
2	rs6732480-TPO	T	51.24	42.5	C	4.807	0.028	1.422 (1.038-1.948)	0.1607
8	rs2741335-EPHX2	G	38.84	30.68	T	4.663	0.031	1.435 (1.033-1.993)	0.1676
11	rs3019593-MTL5	G	11.57	6.818	A	4.531	0.033	1.788 (1.041-3.071)	0.276
2	rs2048727-TPO	A	42.5	34.32	G	4.45	0.035	1.415 (1.024-1.954)	0.1647
17	rs9904720-EPX	A	37.19	29.32	C	4.438	0.035	1.427 (1.024-1.989)	0.1693
5	rs2230303-GPX3	G	1.653	0.2273	T	4.36	0.036	7.378 (0.82-66.39)	1.121
4	rs6833161-SPP1	T	33.88	42.01	C	4.321	0.037	0.7075 (0.5102-0.981)	0.1668
5	rs8177413-GPX3	C	1.653	0.2294	G	4.308	0.037	7.311 (0.8125-65.78)	1.121
8	rs7824574-CLU	A	5.785	10.45	C	4.243	0.039	0.5259 (0.2829-0.9778)	0.3164
22	rs12106549-TXNRD2	T	25.62	18.86	G	4.257	0.039	1.482 (1.019-2.155)	0.1911
17	rs14309-ALOX12	A	7.438	12.5	G	4.186	0.04	0.5625 (0.3222-0.9819)	0.2843
6	rs1293928-IPCEF1	G	30.58	38.41	A	4.174	0.041	0.7063 (0.5057-0.9866)	0.1705
6	rs6905523-GSTA7P	G	26.03	19.32	A	4.143	0.041	1.47 (1.013-2.133)	0.1898
8	rs10087808-OXR1	G	22.31	29.55	A	4.144	0.042	0.6849 (0.4753-0.9871)	0.1864
6	rs35062161-GPX6	T	3.719	1.364	A	4.027	0.044	2.794 (0.9824-7.946)	0.5333
8	rs10109171-OXR1	A	38.43	30.82	G	4.048	0.044	1.401 (1.008-1.947)	0.1678
2	rs2465661-AOX1	T	38.02	45.91	C	3.966	0.046	0.7226 (0.5246-0.9954)	0.1634
7	rs6947821-NOS3	C	29.75	37.27	T	3.902	0.048	0.7128 (0.5091-0.998)	0.1717
8	rs10108813-OXR1	C	22.73	29.77	T	3.907	0.048	0.6938 (0.4823-0.9979)	0.1855
6	rs72944451-GSTA4	T	2.479	68.18	G	3.874	0.049	3.703 (0.9179-14.94)	0.7117
8	rs1503573-OXR1	G	42.15	50	A	3.862	0.049	0.7286 (0.531-0.9996)	0.1614
17	rs11652709-EPX	C	37.6	30.23	G	3.851	0.049	1.391 (1-1.935)	0.1685
20	rs6052780-PRNP	A	1.65	4.545	G	3.848	0.049	0.3529 (0.1192-1.045)	0.5537

## Data Availability

The data used to support the findings of this study are included within the article.
